# Detection of cloned voices in realistic forensic voice comparison scenarios

**DOI:** 10.3389/frai.2025.1678043

**Published:** 2025-11-13

**Authors:** Pedro Univaso, Eugenia San Segundo

**Affiliations:** 1 BlackVOX, Buenos Aires, Argentina; 2 Phonetics Laboratory, Spanish National Research Council, Madrid, Spain

**Keywords:** MFCCs, deepfake, forensic speech evidence, automatic speaker recognition (ASR), acoustic data

## Abstract

Deepfakes and synthetic audio significantly degrade the performance of automatic speaker recognition systems commonly used in forensic laboratories. We investigate the effectiveness of Mel-Frequency Cepstral Coefficients (MFCCs) for detecting cloned voices, ultimately concluding that MFCC-based methods are insufficient as a universal anti-spoofing tool due to their inability to generalize across different cloning algorithms. Furthermore, we evaluate the performance of the HIVE AI-deepfake Content Detection tool, noting its vulnerability to babble noise and signal saturation, which are common in real-world forensic recordings. This investigation emphasizes the ongoing competition between voice cloning and detection technologies, underscoring the urgent need for more robust and generalized anti-spoofing systems for forensic applications.

## 1 Introduction

The emergence of deepfakes and cloned voices presents a significant challenge in the field of forensic voice comparison (FVC). An audio deepfake is a synthetic voice generated by deep learning models, particularly neural networks, that bears an extreme resemblance to a real voice and can therefore be used to clone voices and impersonate a speaker (San Segundo and Delgado, 2025). These synthetic recordings compromise the reliability of automatic speaker identification systems commonly employed in forensic laboratories, as their performance substantially degrades when encountering cloned voices that aim to mask original identities. This problematic scenario underscores a critical need: the ability to identify whether questioned recordings are original or cloned before applying speaker identification systems, thereby ensuring the validity of forensic results. While anti-spoofing systems exist to detect fake or cloned voices, the continuous development of new cloning algorithms creates a competitive landscape, leaving the problem of reliable detection largely unresolved.

In FVC, experts have to compare an unknown voice (belonging to a criminal) to one or more known voices (belonging to suspects). Until now, the main challenges forensic phoneticians faced when undertaking this task —typically required by the court or in collaboration with the police— were the short duration of voice recordings in most cases, or the degradation of their quality due to background noise or the transmission channel, among other factors^1^. Today, the greatest problem has become determining whether a questioned recording is real (i.e., produced by a human) or artificially generated. However, it still remains important to conduct deepfake research with audio samples in the above-mentioned realistic forensic conditions, i.e., channel mismatch, noise and signal saturation, etc. (San Segundo et al., 2019).

Some recent studies examine the current state of voice spoofing detection. For example, Tan et al. (2021) reviewed 172 papers published between 2015 and 2021. They provide a useful taxonomy of the types of attacks identified, the challenges they share, and highlight future directions for research in the field. Meanwhile, Yi et al. (2023), in a more recent study, offer a comprehensive review, identifying key differences between various types of voice deepfakes, describing and analyzing available datasets, acoustic features studied, as well as types of classifiers and evaluation metrics, along with a description of cutting-edge methodological approaches. For each aspect, they discuss foundational techniques, recent developments, and major challenges. In addition, the authors present a unified comparison of representative features and classifiers across different datasets for audio deepfake detection. Their study shows that future research should address the lack of large-scale datasets, the poor generalizability of existing detection methods against unknown spoofing attacks, and the interpretability of detection results.

In terms of acoustic features, MFCC (i.e., the coefficients that make up a Mel-Frequency Cepstrum, MFC), or CQCC (i.e., the coefficients extracted from Constant Q Transform, CQT) are commonly used, with different classifiers; namely, conventional, deep learning and multiple classifiers. These are calculated from frames or windows trying to describe the spectral envelope of sound, as a way of explaining the resonance properties of the vocal tract.

The aim of this paper is twofold: (1) to assess the performance of a state-of-the-art automatic speaker recognition system based on MFCCs^2^ with cloned voices, and (2) to evaluate the performance of a commercial anti-spoofing tool under various forensically-realistic conditions: utterance and channel mismatch, added noise and signal clipping.

- Evaluation across different algorithms, utterances and channels: The probability of detection of genuine and cloned emissions generated by three different algorithms (Eleven Labs, Speechify, Play-ht) and recorded through two different channels (Microphone and WhatsApp) was measured.
- Noise Impact: Detection of genuine and cloned emissions (Eleven Labs) was evaluated under different types of added noise (Music, Babble Noise, White Noise) at varying Signal-to-Noise Ratio (SNR) levels (10, 20, 30, ∞ dB).
- Saturation Impact: The detection performance for genuine and cloned emissions (Eleven Labs) was evaluated across different levels of signal clipping (0, 5, 10, 15 dB amplification, corresponding to no, slight, medium, and high signal clipping).

## 2 Materials and methods

### 2.1 Datasets and evaluation metrics

- DEEP-VOICE dataset (Bird and Lotfi, 2023)^3^. A total of 62 min and 22 s of speech were collected from eight English-speaking public figures, resulting in 5,889 original and 5,889 cloned recordings generated using a convolutional neural network.
- In-the-wild (ITW) (Müller et al., 2022). This dataset presents a balanced mix of both spoofed and genuine speech, sourced from publicly accessible platforms like podcasts and political addresses. The fake clips were created by segmenting publicly available video and audio files that explicitly advertise audio deepfakes. It includes 17.2 h of synthetic audio and 20.7 h of real speech, totaling 31,779 utterances with an average length of 4.3 s. All recordings feature English-speaking public figures, including celebrities and politicians. Recordings with effective durations exceeding 2 s were selected, resulting in a dataset of 24 recordings from 12 speakers (12 genuine and 12 spoofed samples).
- *Ad hoc* dataset. A new database was created to analyze various characteristics of voice cloning, including the influence of different market algorithms, emitted text, and speakers.
  - *Genuine sample generation*: A 66-year-old male Spanish speaker with an Argentinian accent was recorded using a microphone, speaking four utterances: (1) “Hola, te llamo para saber si podés venir mañana para instalar la bomba de agua en la pileta”; (2) “En marzo del año que viene tengo que dictar un curso de Metodología de la Investigación en la Universidad Austral,” (3) “El próximo paso para presentar el proyecto del Instituto Madero a los suizos es ajustar el presupuesto,” (4)"Tengo tiempo, pero no mucho, porque tengo que ir a comprar carbón para el asado de hoy.”
  - *Cloned recording generation*: These original recordings were cloned using three well-known commercial cloning systems: Eleven Labs, Speechify, and Play-ht. The new database was structured according to the following guidelines:
    - Clone of phrase *m* emitting phrase *n* with system *p*: where *m* = [1 to 4], *n* = [1 to 4], and *p* = [Eleven Labs, Speechify, Play-ht].
    - Clone of clone of (1) employing the same system *p*.
    - Clone of phrase *m* emitting phrase *n* with system *p*, varying the system parameters: where *m* = [1 to 4], *n* = [1 to 4], and *p* = [Eleven Labs].

The comparison of voices was performed using FORENSIA (Univaso et al., 2020), an automatic speaker identification system based on the i-vector/PLDA approach, employed in the forensic laboratories of the Supreme Court of Justice and the National Gendarmerie of Argentina. The performance was measured using the Equal Error Rate (EER) metric.

### 2.2 Feature extraction

To assess the potential for predicting cloned recordings, 20 Mel-Frequency Cepstral Coefficients (MFCCs) were analyzed. The DEEP-VOICE dataset included them, but the In-The-Wild dataset they had to be generated using Praat (Boersma and Weenink, 1992). The first MFCC coefficient, which represents the energy of the recording, was excluded, as it was not considered relevant for this analysis. The temporal average of the coefficients was used to represent the long-term spectrum.

A Hamming window of 15 ms was applied every 5 ms. The coefficients were then temporally averaged, excluding the first energy coefficient. Correlation coefficients were used to quantify the spectral variations and similarities between recordings.

### 2.3 Detector performance evaluation

The detection tool selected for this study was HIVE AI Detector (Hive, 2023), which outperformed competing models as well as human expert analysis in an independent research study (Ha et al., 2024). Additionally, HIVE was chosen from among 36 companies to test its deepfake detection and attribution technology in collaboration with the U.S. Department of Defense (Heikkila, 2024).

This tool provides results as a percentage probability that a given speech emission has been cloned. HIVE was evaluated in the present study under these conditions:

- Evaluation across different algorithms, utterances and channels: The probability of detection of genuine and cloned emissions generated by three different algorithms (Eleven Labs, Speechify, Play-ht) and recorded through two different channels (Microphone and WhatsApp) was measured.
- Noise Impact: Detection of genuine and cloned emissions (Eleven Labs) was evaluated under different types of added noise (Music, Babble Noise, White Noise) at varying Signal-to-Noise Ratio (SNR) levels (10, 20, 30, ∞ dB).
- Saturation Impact: The detection performance for genuine and cloned emissions (Eleven Labs) was evaluated across different levels of signal saturation (0, 5, 10, 15 dB amplification, corresponding to no, slight, medium, and high signal clipping).

## 3 Results

### 3.1 Automatic speaker recognition system performance

As can be seen in Table 1, the performance of our automatic speaker identification system decreases substantially when the questioned recordings originate from cloned versions of original voices.

**Table 1:** Equal error rate (EER) results using automatic speaker recognition system FORENSIA and comparing bonafide and spoofed samples in the ITW dataset.

Type of comparison	EER
Genuine vs. Genuine	0.06
Genuine vs. Cloned	0.20
Cloned vs. Cloned	0.20

This issue highlights the need to identify, prior to using these systems, whether the questioned recordings being analyzed are original or cloned. For this purpose, there are deep-fake speech detectors (Nguyen et al., 2025), also known as voice-fake detectors (Tamilselvan and Biswal, 2024) or more generically referred to as audio anti-spoofing detectors (Li et al., 2024). These countermeasures should be employed as a preliminary screening stage before the use of speaker identification systems to ensure their validity. The current challenge lies in the fact that the development of reliable detection algorithms is in constant competition with the advancement of new spoofing or voice cloning algorithms.

It is worth noting that the EER obtained when comparing cloned recordings is similar to that obtained when comparing original and cloned recordings, although the former comparisons do not arise in forensic casework, where there is always control over the reference recordings (known samples).

### 3.2 Cloned voices detection using MFCC features

**Figure 1:**
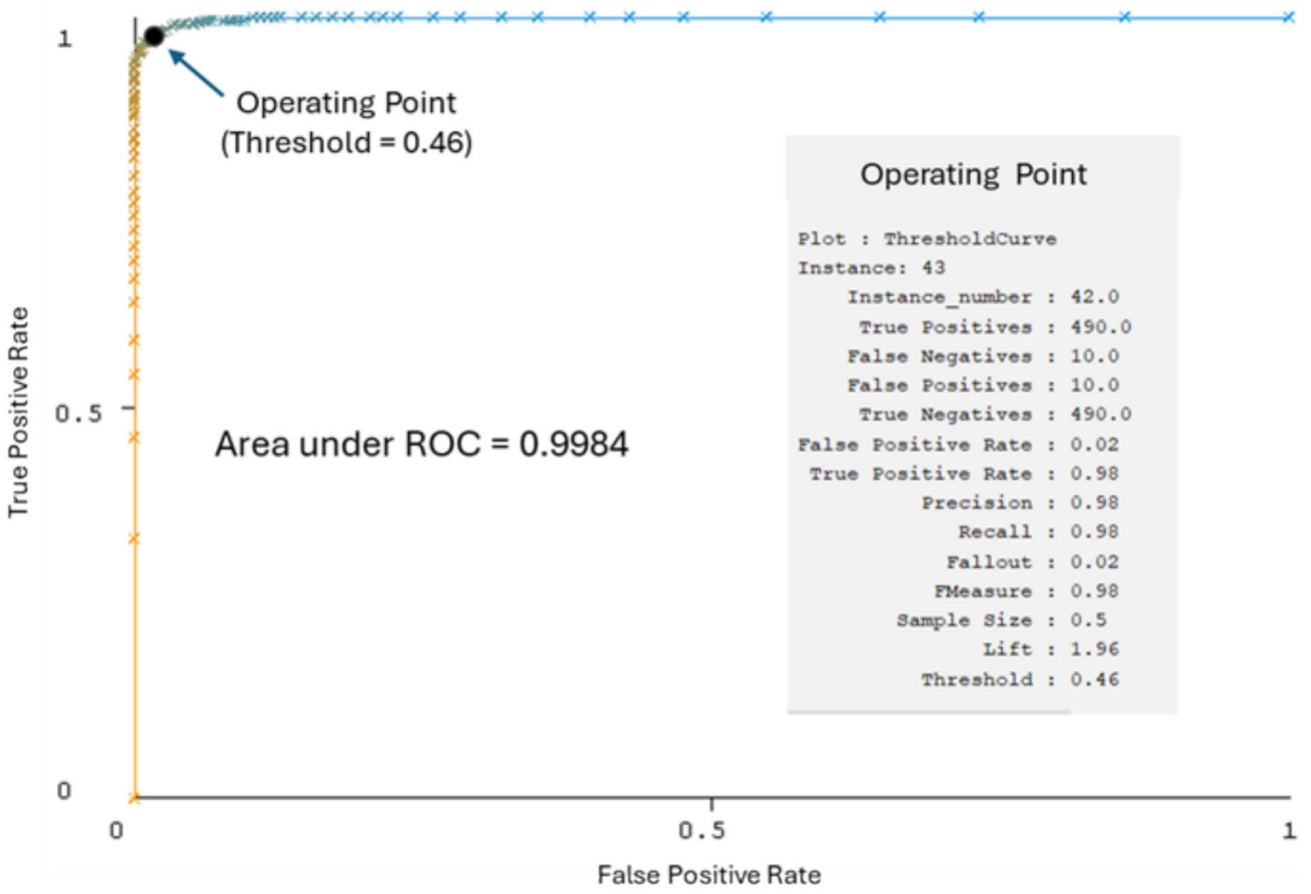
ROC curve of the Random Forest model on the DEEP-VOICE dataset.

A Random Forest model was implemented in the WEKA toolkit (version 3.8.6; Hall et al., 2009). The model was trained using 5,399 genuine and 5,399 cloned samples from the DEEP-VOICE dataset and evaluated on an additional 500 genuine and 500 cloned samples. The results obtained (Figure 1; Table 2) suggest that MFCCs may be a promising tool for detecting cloned voices.

**Table 2:** Performance evaluation of the Random Forest model on the DEEP-VOICE dataset.

Class	Precision	Recall	F-Measure	ROC Area
Cloned	0.98	0.98	0.98	1.00
Genuine	0.98	0.98	0.98	1.00
Average	0.98	0.98	0.98	1.00

**Figure 2:**
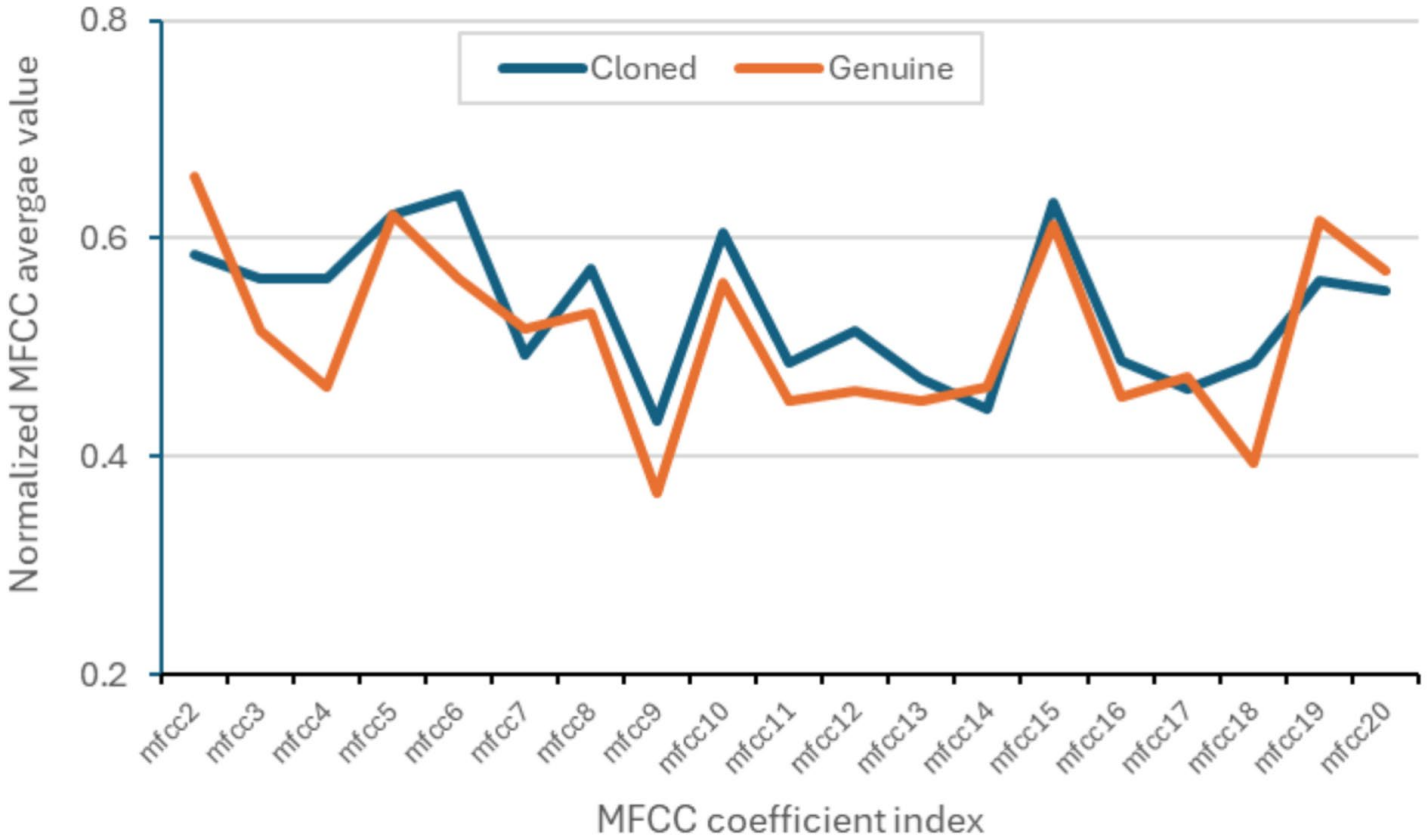
Average values of the MFCC coefficients from 5,889 genuine recordings and 5,889 cloned recordings from the DEEP-VOICE dataset.

Figure 2 shows the differences in MFCC values between original and cloned voices, with an average correlation coefficient of 0.86.

To compare the results reported in Table 2, the trained convolutional neural network model for detecting cloned recordings (DEEP-VOICE) was evaluated on the In-The-Wild (ITW) database. The ITW dataset comprises 12 genuine and 12 cloned samples, including cloned recordings extracted from publicly available video and audio files that explicitly promote audio deepfakes (Table 3).

**Table 3:** Performance metrics of the Random Forest model trained on the DEEP-VOICE dataset and tested on the in-the-wild dataset.

Class	Precision	Recall	F-Measure	ROC Area
Cloned	0.50	0.92	0.65	0.48
Genuine	0.50	0.08	0.14	0.48
Average	0.50	0.50	0.40	0.48

In this case, the precision drops from 0.98 (Table 1) to 0.50. This suggests that the use of different voice cloning algorithms undermines the reliability of MFCC coefficients as predictive parameters. Consequently, they cannot be consistently employed in anti-spoofing systems, contrary to initial expectations. This methodology would only be applicable if the specific cloning algorithm were known and if the model had been previously trained on data generated using that same algorithm. In other words, the MFCC-based approach does not generalize across different types of voice cloning techniques.

**Figure 3:**
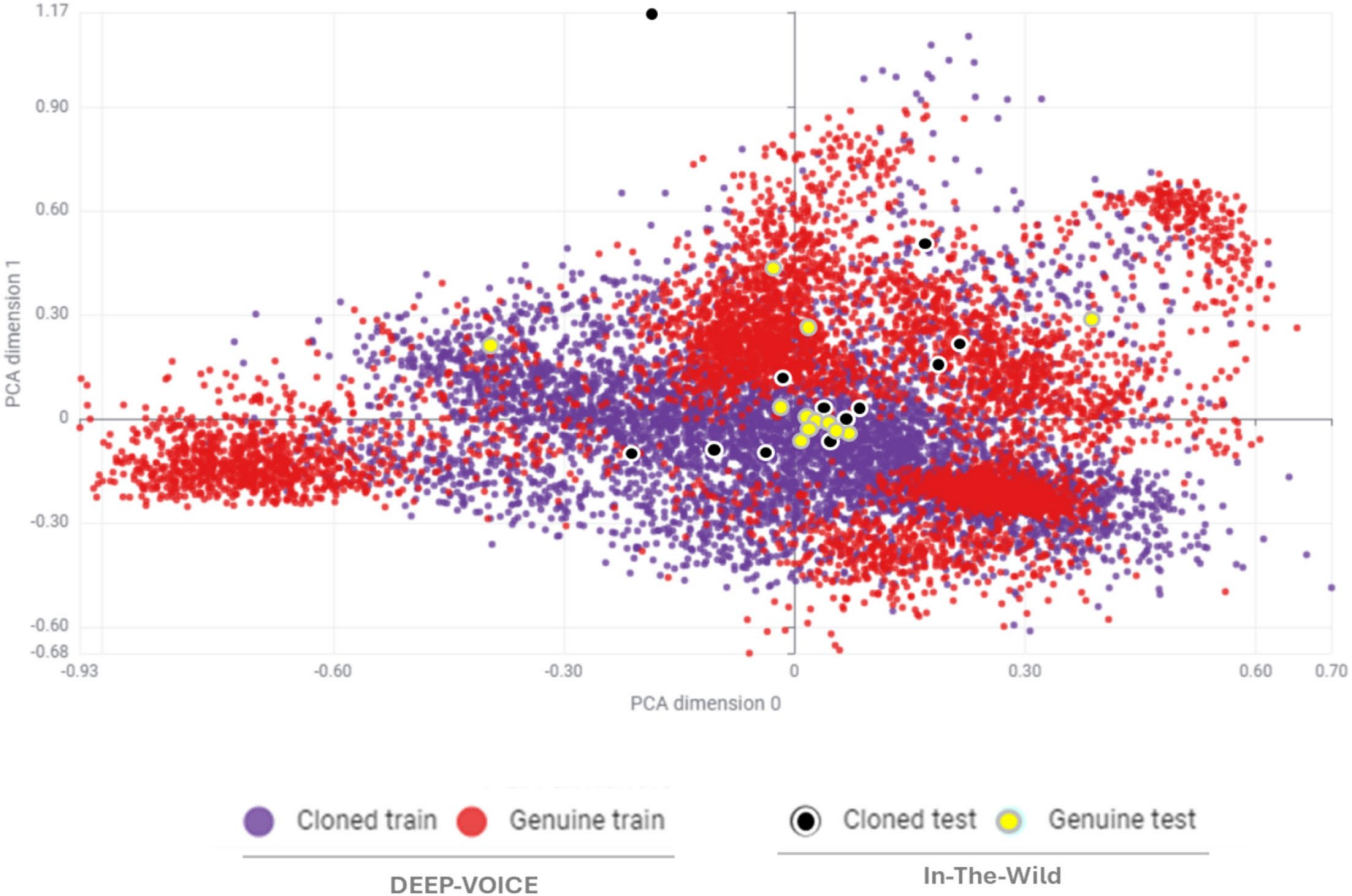
MFCC feature distribution (2D PCA) of genuine and cloned samples from DEEP-VOICE and in-the-wild datasets.

Figure 3 shows the MFCC distributions of DEEP-FAKE and In-The-Wild datasets from genuine and cloned samples, represented in a two-dimensional Principal Component Analysis (2D PCA) plot. It can be observed that the majority of genuine test data fall within the cloned training region, leading to detection errors.

### 3.3 Voice cloning detection performance across algorithms, utterances, channels, noise, and signal clipping

#### 3.3.1 Experiment I: performance across algorithms, utterances, and channels

As shown in Table 4, the results of the HIVE detector for four cloned utterances generated using different algorithms and channels indicate poor performance when the cloned utterance is identical to the genuine utterance (a scenario of limited relevance in forensic cases). On average, the detection likelihood is 0.61; however, it is notably lower when the cloning algorithm used is ElevenLabs. Conversely, when the cloned utterance differs from the genuine one — the scenario of primary forensic interest — the likelihood of detection increases to an average of 0.86. The system correctly identified all four genuine utterances.

**Table 4:** Probability of detection by HIVE of four cloned utterances generated by three cloning algorithms from a genuine utterance recorded through two channels.

Genuine utterance #1		Deepfake detection likelihood (HIVE)			
Cloned algorithm	Channel	Cloned utterance #1	Cloned utterance #2	Cloned utterance #3	Cloned utterance #4
Eleven labs	Microphone	**0.00**	0.99	1.00	0.83
Speechify	Microphone	1.00	1.00	1.00	**0.19**
Play-ht	Microphone	0.99	0.86	0.96	0.99
Eleven labs	WhatsApp	**0.04**	**0.12**	1.00	1.00
Speechify	WhatsApp	1.00	1.00	1.00	1.00
Average		0.61	0.86		

Values in bold mean that those results were not detected as deepfakes.

#### 3.3.2 Experiment II: performance across noise

In this experiment, a cloned utterance was contaminated with three types of noise at different Signal-to-Noise Ratio (SNR) levels, and HIVE was used to detect the type of utterance (cloned or genuine). The results (Table 5) show that the addition of babble noise at low SNR levels masks the detection of the cloned utterance. HIVE correctly identified all four genuine utterances, across the same types of noise and varying SNRs.

**Table 5:** Probability of detection by HIVE of a cloned utterance under different types and levels of noise.

SNR (dB)	Deepfake detection likelihood (HIVE)		
	Music	Babble noise	White noise
10	0.89	**0.00**	0.87
20	0.94	**0.05**	0.98
30	0.97	0.97	0.96
∞	1.00	1.00	1.00
Average	0.95	0.50	0.95

Values in bold mean that those results were not detected as deepfakes.

#### 3.3.3 Experiment III: performance across signal clipping

In this experiment, a cloned utterance was amplified to generate different levels of clipping in order to analyze their effect on the HIVE deepfake detector. The results (Table 6) show that only moderate and high levels of clipping affect the detection of cloned utterances. HIVE correctly identified all four genuine utterances, under the same levels of clipping.

**Table 6:** Probability of detection by HIVE of a cloned utterance under different types and levels of clipping.

Signal level (dB)	Clipping	Deepfake detection likelihood (HIVE)
0	No	1.00
5	Mild	0.86
10	Moderate	**0.52**
15	Severe	**0.00**

Values in bold mean that those results were not detected as deepfakes.

## 4 Discussion

This study highlights a critical vulnerability in current forensic voice analysis: the substantial reduction in performance of automatic speaker identification systems when confronted with cloned voices. This underscores the urgent need for robust pre-identification of synthetic speech. Our initial exploration into MFCC-based detection showed promising precision for a specific dataset. However, a key and novel finding was that this MFCC-based model failed to generalize across different cloning algorithms, with precision dropping by 51% when evaluated on a different database. This demonstrates that while MFCCs can discriminate between a known original and a known clone, they are insufficient as a universal anti-spoofing parameter when the cloning algorithm is unknown. This is a significant insight, as it suggests that simply relying on typical acoustic features used in automatic speaker recognition without knowledge of the cloning generation process is inadequate for the rapidly evolving landscape of voice cloning.

A significant part of our study focused on evaluating the HIVE AI-generated content detection tool, a commercial solution, across different forensically realistic conditions. Our findings indicate that HIVE achieves strong performance (precision = 0.86) on commercial voice cloning algorithms in ideal conditions and demonstrates high accuracy in differentiating original from cloned voices. The novelty here lies in the comprehensive stress-testing of this tool against various types of noise and signal degradation. Crucially, HIVE’s performance degraded substantially in the presence of babble noise and medium to high signal saturation. This specific identification of vulnerabilities is critical for understanding the real-world applicability of such tools in noisy or compromised forensic recordings.

While recent studies have evaluated how well anti-spoofing systems perform under forensically realistic degradations such as channel and utterance mismatch, added noise, compression, and reverberation (e.g., Gomez-Alanis et al., 2018; Cohen et al., 2022; Zhang et al., 2021), to the best of our knowledge there are no anti-spoofing studies that run controlled experiments isolating signal clipping (digital clipping) as a primary variable. Most robustness studies have focused on added noise (various SNRs), reverberation/room impulse responses, channel effects (device impulse responses), codecs/compression (telephony/VoIP), and utterance/attack mismatch.

For instance, Gomez-Alanis et al. (2018) focused on noise robustness. They proposed noise-aware training and soft-masking to improve robustness to additive noise and reverberation. Zhang et al. (2021) evaluated cross-dataset channel mismatch and found that when systems were trained on ASVspoof2019LA and tested on other datasets, EERs degraded drastically. Cohen et al. (2022) is a targeted data-augmentation study that shows that compression augmentation and channel augmentation substantially reduce EER on DeepFake and Logistic Access tasks. This study also reports large relative improvements over unaugmented baselines when simulating codecs/telephony/bandwidth degradations.

All in all, our findings are in line with the above-discussed studies, which consistently show that performance deteriorates sharply when models trained on clean or matched conditions are tested under realistic mismatches.

The primary limitation of the MFCC-based detection approach is its lack of generalization across different voice cloning algorithms. This means that for forensic applications, a model trained on MFCCs would require prior knowledge of the specific cloning algorithm used, along with sufficient training data for that algorithm, which is often unfeasible in real-world scenarios.

Another significant shortcoming lies in the robustness of current detection tools. While the HIVE detector shows promise for commercial algorithms, its vulnerability to babble noise and signal saturation is a critical concern for forensic evidence, which frequently originates from environments with background noise or has undergone various forms of signal degradation. For example, signal degradation typically arises through recording or transmission via channels such as WhatsApp, as well as social networks, where deepfakes exploit these types of signals to mislead AI detectors. This suggests that even advanced commercial tools are not yet sufficiently robust for all real-world forensic applications. Furthermore, the Eleven Labs’ own detector’s ineffectiveness against clones from other algorithms (as promoted on their website^4^) highlights the prevalent issue of algorithm-specific detection, which is impractical for general forensic use where the source of a clone is typically unknown.

## 5 Conclusions and directions for future research

This research significantly advances our current understanding of voice deepfakes by providing empirical evidence that the challenge of detecting cloned voices is indeed “far from resolved” due to the continuous competition between cloning and detection algorithms. It reinforces the urgent necessity for anti-spoofing measures to be implemented as a preliminary step in forensic voice analysis to ensure the validity of subsequent speaker identification processes.

The comprehensive evaluation of a commercial tool like HIVE provides a valuable benchmark for the state-of-the-art in AI-generated content detection. By precisely identifying its strengths (detecting known commercial clones under clean conditions) and weaknesses (susceptibility to babble noise and saturation), this study offers concrete targets for future research and development in this domain. It confirms that while progress is being made, current solutions still fall short of the robust, universal detection capabilities required for challenging forensic contexts.

Given the limitations identified, future research must prioritize the development of anti-spoofing systems that exhibit strong generalization capabilities across a wide and unknown range of current and future cloning algorithms. This may require moving beyond traditional acoustic features like MFCCs to explore more complex, perhaps less intuitive, markers of synthetic speech. Deep learning architectures, particularly those capable of learning robust representations from raw audio or spectro-temporal patterns, could be promising avenues. These models might be able to identify subtle, pervasive artifacts introduced during the cloning process, irrespective of the specific algorithm employed.

Furthermore, research should focus on enhancing the robustness of detection systems against real-world acoustic challenges, specifically addressing the adverse effects of various noise types (especially babble noise) and signal degradation (like saturation). This could involve incorporating noise reduction techniques as a pre-processing step or training models on highly diverse datasets that include speech corrupted by various types and levels of noise and distortion.

It is also pertinent to speculate on the potential for multi-modal detection approaches. Combining acoustic analysis with other indicators, such as linguistic patterns, semantic consistency, or even analyzing the meta-data or source of the recording could provide a more holistic and robust detection framework. This would require interdisciplinary collaboration beyond traditional speech forensic scientists. Finally, further investigation into the unique “fingerprints” left by specific cloning algorithms at a very granular level might lead to the development of an ensemble of specialized detectors. Such an ensemble could attempt to identify the most probable cloning algorithm, and then apply algorithm-specific models, thereby improving overall accuracy when the algorithm can be inferred. However, the overarching goal remains a universal detector that does not rely on such inferences.

## Data Availability

The raw data supporting the conclusions of this article will be made available by the authors, without undue reservation.

## Footnotes

^1^Poor recording quality also affects the field of forensic speech enhancement (FSE). See Mawalim et al. (2024) for a recent discussion of the quality factors influencing speech recordings used as evidence in criminal trials.

^2^The motivation for analyzing MFCC-based features in relation to ASR performance with cloned voices lies in the fact that MFCCs remain a core acoustic representation in most ASR systems. Because cloned or synthetic voices often reproduce the spectral envelope of natural speech imperfectly, examining how MFCC-based representations capture or amplify these differences is crucial for assessing the robustness of ASR systems to synthetic inputs.

^3^The data used in this study is available from: https://www.kaggle.com/datasets/birdy654/deep-voice-deepfake-voice-recognition.

^4^ https://elevenlabs.io/es/ai-speech-classifier
